# Extraordinary Capability for Water Treatment Achieved by a Perfluorous Conjugated Microporous Polymer

**DOI:** 10.1038/srep10155

**Published:** 2015-05-14

**Authors:** Rui-Xia Yang, Ting-Ting Wang, Wei-Qiao Deng

**Affiliations:** 1 State Key Laboratory of Molecular Reaction Dynamics, Dalian National Laboratory for Clean Energy, Dalian Institute of Chemical Physics, Chinese Academy of Sciences, 457 Zhongshan Road, Dalian 116023, R. P. China

## Abstract

Oils, organic solvents, dyes, and heavy metal ions are primary pollutants in water resources. Currently, no sorbent material can effectively remove these types of pollutants simultaneously. Here we report a perfluorous conjugated microporous polymer with superhydrophobicity and a large surface area, which exhibits outstanding adsorption capacities, kinetics, and recyclability for a wide range of organic solvents, oils, dyes, and heavy metal ions. The adsorption capacities of this polymer, 1376.7 mg g^−1^ for Congo red, 808.2 mg g^−1^ for Pb(II) and 303.2 mg g^−1^ for As(V), are higher than the adsorption capacities of any previously described porous materials. Our theoretical calculation reveals that the superior properties of this polymer are due to fluorination and triple bonds within the polymer. A benchmark experiment indicates that this polymer can efficiently remove these pollutants simultaneously. Application of this polymer may lead to the development of next-generation reusable and portable water purification appliances.

More than one-third of the human population remains without access or with only limited access to sanitary and safe drinking water despite major efforts to develop effective, economical, and robust methods for water purification^1^. The effective removal of chemical contaminants, including oils/organic solvents, heavy metal ions, and dyes, from water is one of the major challenges^2^,^3^. Currently, adsorption is regarded as the most favourable method for water cleansing, but common adsorbents, including activated carbon^4^, zeolites^5^ and natural fibers^6^, suffer from low adsorption capacities, poor selective sorption and an unsatisfactory regeneration ability. A number of advanced adsorbents, including nanostructured metal oxides^7^,^8^, carbon nanotubes^9^,^10^, porous BN nanosheets^11^ and porous graphene^12^, among others^13^,^14^, have been developed to overcome these shortcomings. However, to the best of our knowledge, all of the reported adsorbents can only significantly adsorb one or two of the three types of pollutants mentioned above. Therefore, a versatile adsorbent that can efficiently remove all three types of pollutants would be highly desirable for the development of a portable and economical water purification appliance for the people without access to clean water resources.

Conjugated microporous polymers (CMPs), first reported in 2007, have attracted much research attention as a result of their finely tunable microporosity, large specific surface areas, high chemical and thermal stability and extended π-conjugation^15^. The breadth of applications for CMPs has been rapidly increasing and these applications include gas storage^16^,^17^, catalysis^18^,^19^,^20^, photoluminescence^21^, light-harvesting networks^22^, electronics^23^, photovoltaics^24^, and supercapacitors^25^. We have previously reported on the superhydrophobicity of conjugated microporous polymers, and their value in the field of oil/water separation^26^.

Enlightened by that the fluorination can enhance the hydrophobicity of conjugated microporous polymers^27^, in this work we synthesized a perfluorous conjugated microporous polymer, with a water contact angle of 160°, which can efficiently adsorb heavy metal ions, toxic organic solvents and waste oils, and chemical dyes. The capacity, kinetics, recovery, regeneration, and recyclability in the adsorption process of this polymer were significant. Moreover, the extraordinary adsorption capacities for the three types of pollutants were among the best seen for any previously described microporous sorbents. In addition, the used polymer can be recycled by a simple washing method.

## Results

### Synthesis

**Figure 1:**
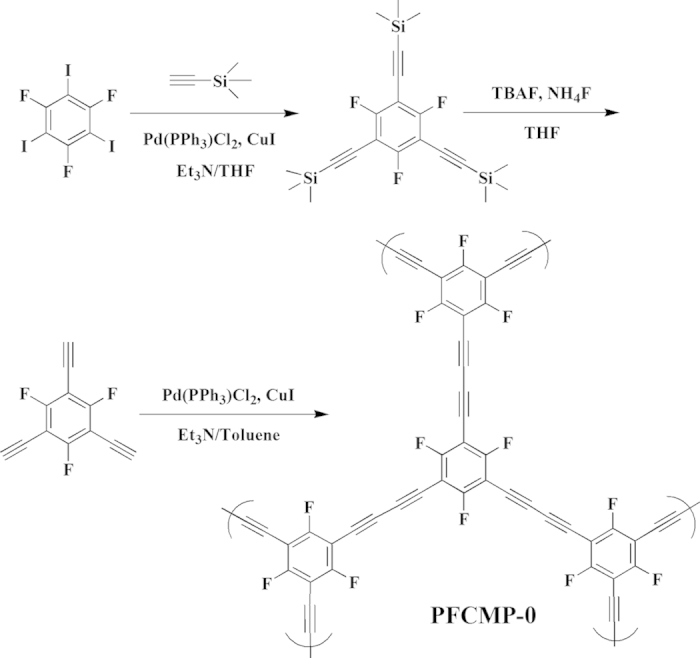
The synthesis route of PFCMP-0.

A perfluorous conjugated microporous polymer, named PFCMP-0, was synthesised via a Pd(II)/Cu(I)-catalysed homocoupling polymerisation of 1,3,5-trifluoro-2,4,6 - triethynylbenzene, wherein all of the hydrogen atoms on the benzene ring are substituted with fluorine atoms. The synthesis pathway is shown in Fig. 1, with details shown in the Supporting Information. Similar to other CMPs, PFCMP-0 was insoluble in all of the tested solvents. The structure of PFCMP-0 was characterised at the molecular level by ^1^H-^13^C CP/MAS NMR. The solid-state NMR spectrum is shown in Supplementary Fig. S1. Besides, HCMP-1^26^ constructed by 1,3,5 - triethynylbenzene units was also synthesized for comparison.

### Characterization

**Figure 2:**
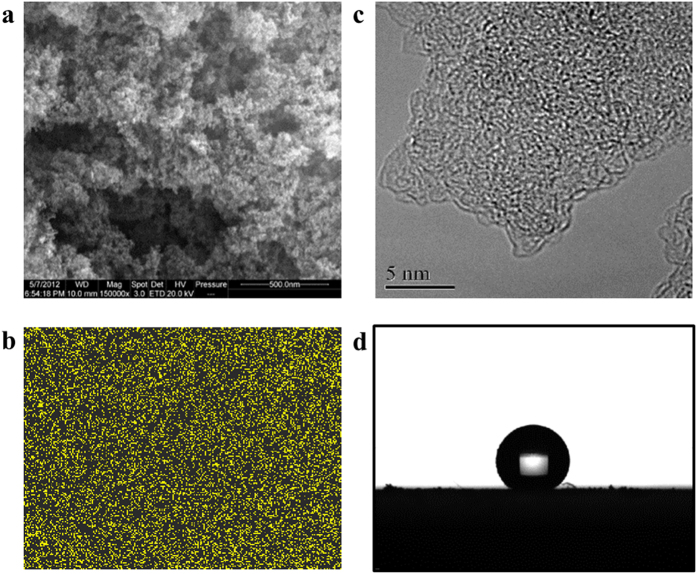
Structural and material properties of PFCMP-0. (**a**) SEM micrograph of PFCMP-0; (**b**) Corresponding EDX F mapping; (**c**) HR-TEM micrograph of PFCMP-0; (**d**) The contact angle with a water droplet.

Thermogravimetric analysis (TGA, Supplementary Fig. S2) shows that PFCMP-0 possesses a high thermal stability, with the thermal decomposition temperature above 300 °C, which was expected because fluorination typically enhances the thermal stability of materials. The field emission scanning electron microscopy (FE-SEM) images reveal that the PFCMP-0 network morphologically consists of agglomerated particles with porous features (Fig. 2a). These data along with the energy dispersive X-ray spectrometer (EDX) analysis (Fig. 2b and Supplementary Fig. S3) indicate that the fluorine is homogeneously distributed throughout the polymer network. Moreover, the finely ordered microporosity of PFCMP-0 can also be observed in the high-resolution transmission electron microscope (HR-TEM) image, as shown in Fig. 2c and Supplementary Fig. S4.

We also investigated the porosity of PFCMP-0 using N_2_ gas sorption isotherms according to the IUPAC classifications. The nitrogen isotherms of PFCMP-0 (Supplementary Fig. S5) at 77 K indicate that the material is microporous. The small increase in N_2_ uptake at high relative pressures (P/P_0_ > 0.8) in the adsorption isotherms may stem from interparticulate porosity of the sample. The Brunauer-Emmett-Teller (BET) surface area, which was calculated using the BET equation, was 901 m^2^ g^-1^ for PFCMP-0. The high surface area is ascribed to the three-dimensional micropores in the net structure caused by the rigid skeleton of the alkyne monomer. Analysis of the porous properties shows that the overall micropore volume of PFCMP-0 was 0.39 cm^3^ g^-1^, and the total pore volume of PFCMP-0 was up to 2.34 cm^3^ g^-1^ (calculated by BJH (Barrett-Joyner-Halenda) method), which was significantly higher than many other microporous materials^28^. It can be concluded from the pore size distribution curves (Supplementary Fig. S5) that the compound is primarily composed of micropores (pore sizes less than 2 nm).

Water contact angle (CA) measurements were performed to determine the hydrophobic properties of the materials. PFCMP-0 exhibits surface superhydrophobicity with a water CA of 160°, as shown in Fig. 2d. The superhydrophobicity of PFCMP-0 resulted from the cooperation of both of the microporous morphological structures and the introduction of fluorine atoms, which facilitate surface hydrophobicity^27^. Although superhydrophobic materials with water CA values larger than 160° have been reported in previous studies^29^,^30^, they were typically obtained by complicated and expensive fabrication methods that modify and roughen the surface, which hampers their wide-range applications. However, PFCMP-0 is a bulk material that can be synthesized using a one-step process without any further surface treatment. Hence, as one of the most hydrophobic bulk materials reported thus far, PFCMP-0 promises abundant potential applications because of its facile synthesis procedure.

### Removal of organic solvents and oils

A number of porous materials that can be used for the removal of chemical pollutants, particularly toxic organic solvents and waste oils, have been reported previously. Calcagnile *et al.*^31^ used polyurethane (PU) foam with the surface modified by submicrometer polytetrafluoroethylene (PTFE) particles and colloidal superparamagnetic iron oxide nanoparticles to separate oils from water. Huang *et al.*^32^ fabricated graphene capsules with effective adsorption oil properties and illustrated a correlation between adsorption capacity and surface area. In 2013, Ma *et al.*^33^ synthesized a porous polymer, named PCF-1, with a large surface area of 1,300 m^2^ g^-1^; its uptake capacities for cyclohexane and gasoline were as high as 2,510 wt% and 2,050 wt%, respectively. Subsequently, Lei *et al.*^11^ reported that the adsorption amount of porous boron nitride (BN) nanosheets can be as high as 33 times their weight.

**Figure 3:**
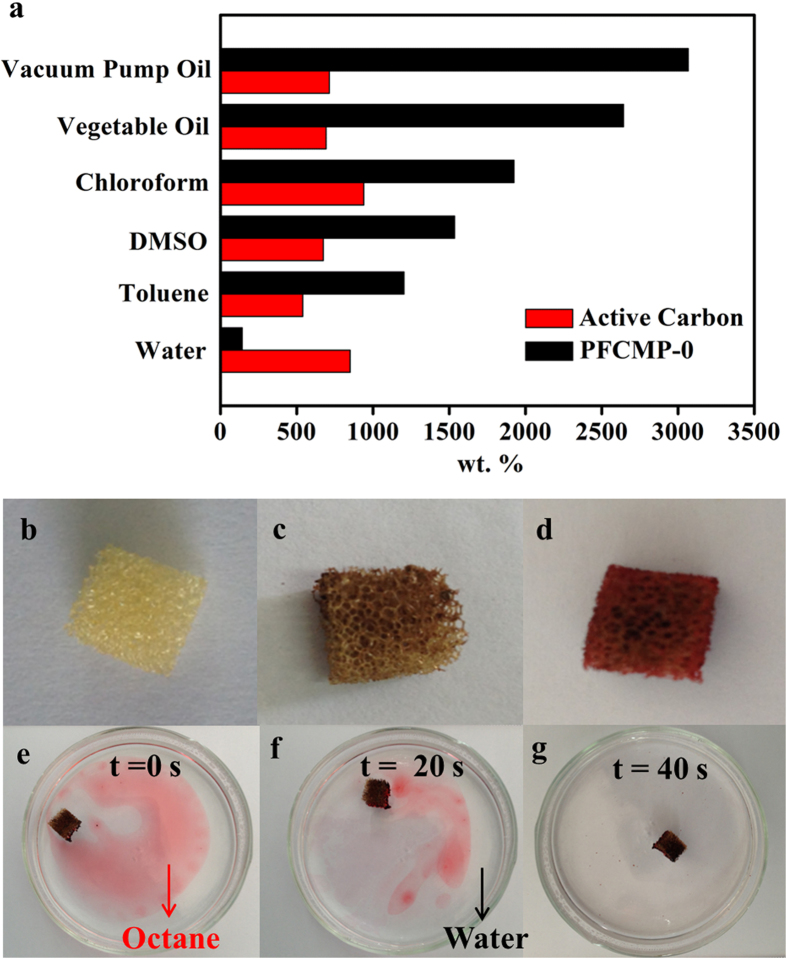
Adsorption properties of PFCMP-0 for solvents and oils. (**a**) Adsorption capacities for different solvents and oils. (**b**) Image of the untreated sponge (0.6 × 0.6 × 0.6 cm). (**c**) Image of the PFCMP-0 treated sponge before adsorption. (**d**) Image of the treated sponge after adsorption. (**e**-**g**) Snapshot images during octane (dyed by red oil o) adsorption.

Because of its superhydrophobicity and porous properties, PFCMP-0 displayed excellent behaviour in adsorbing oils and organic solvents in water. As shown in Fig. 3a, the uptake values of vacuum pump oil, vegetable oil, chloroform, dimethylsulfoxide and toluene were over the range of 1,200 to 3,000 wt%. However, the adsorption capacity of PFCMP-0 for water was only 142 wt%, which is significantly less than that of active carbon (850 wt%). This result was due to the hydrophilicity of active carbon with a water CA of 0° (Supplementary Fig. S6). Noticeably, the adsorption capacities of vacuum pump oil and vegetable oil can be up to 30 and 26 times as much as the weight of PFCMP-0, respectively. These uptake values approximate those of the porous BN nanosheets mentioned above and are significantly higher than those of commercial activated carbon, HCMP-1^26^ (1,100 wt% for pump oil and 1,000 wt% for vegetable oil), nanowire membranes^8^ (2,000 wt% for motor oil), sponges^34^ (1,900 wt% for lubricating oil) and macroscopic multifunctional graphene-based hydrogels^12^ (1,500 wt% for gasoline). Although the uptake capacities of PFCMP-0 were significantly lower than those of macroporous materials such as carbon nanofiber (CNF) aerogels^35^,^36^ (100-320 times), activated carbon-coated sponges^37^ (25-100 times) and nitrogen-doped graphene frameworks^38^ (200-600 times), they are among the best seen among microporous materials. The excellent performance of PFCMP-0 in adsorbing organic solvents and oils is attributed to its outstanding porous properties and superhydrophobicity. The comparison between PFCMP-0 and other materials has been listed in Supplementary Table S1.

To further understand the adsorption kinetics involved, a sponge treated (Fig. 3b–d) with PFCMP-0 was used to trace the adsorption process of octane dyed with red oil o. When the treated sponge was placed on the octane thin film (Fig. 3e–g), the dyed octane was removed almost completely after only 40 s, revealing fast adsorption kinetics. The saturated sponge floated on the clean water surface, and no water uptake was observed, indicating its excellent adsorption selectivity.

More importantly, PFCMP-0 with organic solvents adsorbed can be easily regenerated after extraction of the solvents to avoid secondary pollution. In addition, oil-treated PFCMP-0 can be reused after being immersed in methanol for 10 min and then dried in a vacuum. The uptake capacities of PFCMP-0 did not decrease after 11 regeneration times, which is shown in Supplementary Fig. S7.

### Removal of dyes

**Figure 4:**
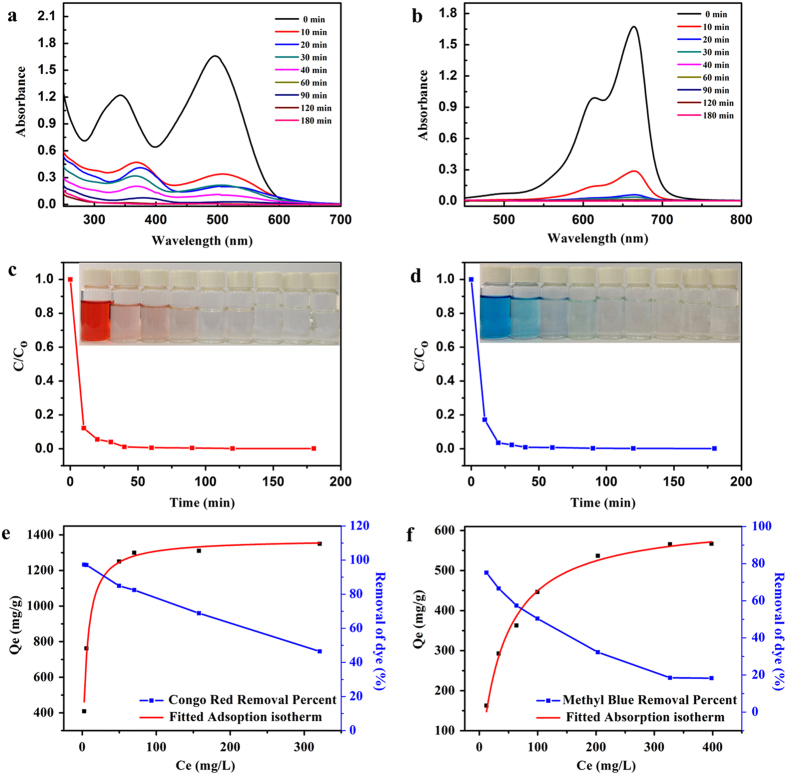
Dye adsorption properties of PFCMP-0. (**a**,**b**) UV–vis adsorption spectra of the CR and MB anhydrous solutions after being treated at different intervals. The initial concentrations of the CR and MB solutions are 100 mg L^−1^ and 25 mg L^−1^, respectively. (**c**,**d**) The adsorption rates of CR and MB adsorption. The insets show the corresponding images. (**e**,**f**) The adsorption isotherms and percentage removal of CR and MB as a function of their equilibrium concentrations.

In addition to organic solvents and oils, PFCMP-0 is also useful for chemical dyes, such as Congo red (CR) and Methyl Blue (MB), which are considered primary sources of pollution in water resources. A UV-vis spectrophotometer was employed to trace the adsorption behaviours of PFCMP-0 according to changes in the intensities of the maximum absorption wavelengths of Congo red and Methyl Blue at 498 nm and 665 nm, respectively. Figure 4a,b shows the absorption spectra at different time intervals of CR and MB aqueous solutions after they were treated with PFCMP-0. It can be observed that the characteristic bands of CR and MB become increasingly weaker with increasing treatment time, and both of the bands were completely removed after 3 h. As shown in Fig. 4c,d, more than 80% of the dyes were adsorbed by PFCMP-0 after 10 min, which accounted for the fast adsorption kinetics of PFCMP-0 for dyes. The inset figures show the corresponding camera images of the dye solution over time. The colours of the dyes become increasingly lighter and finally fade after 3 h. The adsorption isotherms fitted by the Langmuir model are given in Fig. 4e,f. It can be concluded that the maximum adsorption capacities of CR and MB are 1376.7 mg g^-1^ and 629.1 mg g^-1^, respectively, which are significantly higher than for other materials, such as active carbon^39^ (500 mg g^-1^ for CR and 400 mg g^-1^ for MB), CNF-280^40^ (603.43 mg g^-1^ for MB), carbon nanotubes^41^ (882 mg g^-1^ for CR), BN nanosheets^11^ (782 mg g^-1^ for CR and 313 mg g^-1^ for MB), BNHSs C^42^ (116.5 mg g^-1^ for MB) and α-FeOOH hollow spheres^43^ (275 mg g^-1^ for CR). To be compared analogically, the adsorption experiment of HCMP-1 for Congo red was carried out and the results were shown in Supplementary Fig. S8. The maximum adsorption capacity of HCMP-1 for CR is 1065 mg g^-1^, which is much lower than that of PFCMP-0. To our knowledge, the uptake capacity of CR in this study is the highest value to date. Supplementary Table S2 compares PFCMP-0 with other materials.

Furthermore, when PFCMP-0 has been used to adsorb dyes it can be regenerated by washing with hot methanol and drying in a vacuum. The regenerated PFCMP-0 can be reused to adsorb dyes without an obvious reduction in the uptake capacities after 11 cycle times, as shown in Supplementary Fig. S9. Then the methanol was extracted by vacuum distillation and the dyes were recycled.

### Removal of heavy metal ions

**Figure 5:**
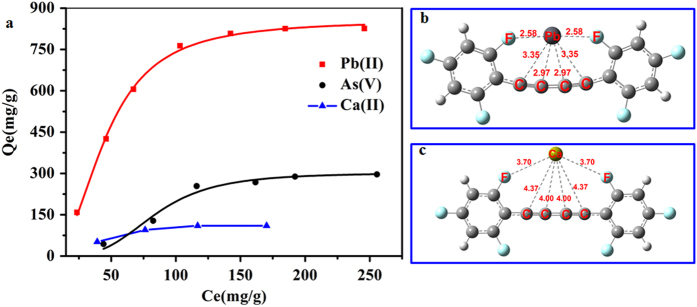
Adsorption properties of PFCMP-0 for metal ions. (**a**) Adsorption isotherms. (**b**) A model of the combination state between the PFCMP-0 and Pb(II). (**c**) A model of the combination state between the PFCMP-0 and Ca(II). The marked values represent the distances between PFCMP-0 atoms and cations.

To further investigate the capture capacities of cationic pollutants in water cleaning, PFCMP-0 was dropped into aqueous solutions containing Pb(II), As(V), and Ca(II) ions, respectively. All of the aqueous solutions of the metal ions used in the adsorption experiments were prepared at different initial concentrations by dissolving the corresponding inorganic compounds into deionised water. Because of its superhydrophobicity, PFCMP-0 floated on the surface of the metal ions water solution without stirring. However, under stirring condition, a uniform dispersion has been formed as shown in Supplementary Fig. S10 because the polymer particles are small enough (several micrometers in sizes observed from SEM images, Supplementary Fig. S4). Thus the metal ions in water can make contact with the polymer and have full access to the pores of the polymer. Figure 5a gives the adsorption isotherms of the three metal ions mentioned above, of which the first two are considered particularly poisonous elements in water. It can be seen that the maximum uptake capacities for Pb(II) and As(V) are 826.1 mg g^-1^ and 303.2 mg g^-1^, respectively. The adsorption capacity values of these two ions are significantly higher than those of previously reported materials, such as active carbon^44^ (21.8 mg g^-1^ for Pb(II)), carbonaceous nanofibre membranes^40^ (423.7 mg g^-1^ for Pb(II)), macroscopic multifunctional graphene-based hydrogels^12^ (373.8 mg g^-1^ for Pb(II)), urchin-like α-FeOOH hollow spheres^43^ (80 mg g^-1^ for Pb(II) and 58 mg g^-1^ for As(V)), ceria hollow nanospheres^45^ (9.2 mg g^-1^ for Pb(II) and 22.4 mg g^-1^ for As(V)), Fe_3_MOSF^46^ (248 mg g g^-1^ for As(V)), as well as CAS3^47^ (152.74 mg g^-1^ for Pb(II)). A comparison of the Pb(II) and As(V) absorption capacities of PFCMP-0 and other materials are listed in Supplementary Table S3 and Supplementary Table S4, respectively. To the best of our knowledge, these uptake values are the highest seen for adsorbent materials reported so far. By contrast, the maximum adsorption of Ca(II) is only 110 mg g^-1^, which is significantly lower than that of Pb(II) and As(V). When the concentration of calcium is 50 mg L^−1^, the amount adsorbed by PFCMP-0 is negligible, which illustrates another significant advantage of PFCMP-0 for water treatment: PFCMP-0 adsorbs toxic metal ions without adsorbing nutritional ions.

Moreover, when PFCMP-0 has adsorbed heavy metal ions it can be easily regenerated and recycled. The used polymer powder was collected by centrifugal filtration and the desorption of the metal ions can be implemented by adding the treated adsorbents to 2 M hydrochloric acid, stirred overnight at room temperature. When the initial concentration of the Pb(II) ion is 50 mg L^−1^, the adsorption capacity of regenerated and recycled PFCMP-0 is 133.97 mg g^−1^ after eleven cycles, representing no significant change compared with 158.58 mg g^−1^ for fresh PFCMP-0, as shown in Supplementary Fig. S11.

### Simultaneous removal of the primary pollutants

An adsorption experiment was performed to provide evidence that PFCMP-0 can simultaneously adsorb the three types of primary pollutants mentioned above. For example, consider a solution in which the concentration of CR and the Pb(II) ion are 10 mg L^−1^ and 5 mg L^−1^, respectively, and the ratio of the volume of toluene added to the solution is 0.1% (with a concentration of 866 mg L^−1^). As given in Supplementary Fig. S12, after adsorption, the absorbance intensity of the characteristic band of CR at 498 nm decreased to nearly zero, which demonstrated that nearly all of the dye was adsorbed. The concentration of the remaining dye in the solution was 0.09 mg L^−1^, as calculated by Lambert-Beer’s law. The concentration of the residual toluene in the solution was determined by Gas Chromatograph-Mass Spectrometer-computer (GC-MS), and the result (Supplementary Fig. S13) showed that there was nearly no toluene left in the solution after absorption. In addition, the concentration of the Pb(II) ion unadsorbed was 0.009 mg L^−1^, as detected by an Inductively Coupled Plasma Emission Spectrometer (ICP). All of these results indicate the unparalleled ability of PFCMP-0 to simultaneously adsorb pollutants for water treatment.

## Discussion

The adsorption capacities of PFCMP-0 for organic solvents and oils are significantly better than those for active carbon by nearly 4 times (Fig. 3a). This result indicates that the three-dimensional stereochemical structure of PFCMP-0 plays an important role in the adsorption process. In addition, the water contact angle of active carbon is nearly 0°, indicating that the active carbon is hydrophilic; therefore, active carbon is more prone to adsorb water than organic solvents and oils. There are two reasons for the high uptake capacities of PFCMP-0: a) the superhydrophobicity and large surface area of PFCMP-0 enhance the adsorption of oil molecules on its surface; and b) the large pore volume of PFCMP-0 enables the pores inside the PFCMP-0 framework to be filled with more solvent or oil molecules.

Infrared Spectroscopy (IR) was used to trace the dyes adsorbed on the polymer. Compared with the FT-IR spectra of PFCMP-0 in Supplementary Fig. S14, the adsorption bands caused by the C-N vibration of the aromatic amine in the structure of the Congo red molecule at approximately 1200 cm^−1^ were clearly observed after the PFCMP-0 adsorbed the dyes, which confirmed that the chemical dyes were adsorbed inside PFCMP-0. As mentioned above, the adsorption capacities of PFCMP-0 for chemical dyes are significantly higher than for other porous materials, primarily because of the large surface area of PFCMP-0. Moreover, the large amount of fluorine atoms distributed on the edges of the polymer network structure are highly electronegative and might provide powerful adsorption sites to attract chemical molecules such as dyes.

The adsorption capacities of PFCMP-0 are significantly higher than other materials with nanostructures, especially for toxic cations such as Pb(II) and As(V). The extraordinary adsorption performance of PFCMP-0 is derived from the following two factors: a) excellent pore properties, including a large surface area and an enormous pore volume; and b) interaction between the covalently bonded fluorine atoms and metal cations, which has been discussed in detail^48^,^49^,^50^. To further understand the mechanism of capturing heavy metal ions employed by PFCMP-0, we obtained quantum mechanics calculations performed with the LanL2DZ basis set using the Gaussian 09 software package^51^ based on the DFT method. We also incorporated considerable solvent effects^52^ into the calculation and estimated the solvation energies in terms of the PCM model^53^ with water as a solvent. The results from the calculation provide a model of the combination state between the PFCMP-0 segment and the metal ions in the adsorption process. Figure 5b shows that an extended π-conjugation forms between the charge distribution around the plane of the benzene rings and the triple bonds in the terminal alkynes, forming a π-electron cloud, which facilitates a noncovalent interaction between the π-electron cloud and Pb(II). Meanwhile, the fluorine atoms in the polymer network can function as donor groups, attracting Pb(II), because of their high electronegativity. These two effects contribute to the powerful adsorption ability of PFCMP-0 to Pb(II). The marked values represent the distances between Pb(II) and atoms of PFCMP-0. In addition, these effects exist between Ca(II) and PFCMP-0, as shown in Fig. 5c. However, the interaction between Ca(II) and PFCMP-0 is significantly weaker. The calculated binding energy between Pb(II) and PFCMP-0 is 10.76 kcal mol^−1^, which is significantly higher than that between Ca(II) and PFCMP-0 (0.47 kcal mol^−1^). Therefore, PFCMP-0 shows a much stronger adsorption ability for Pb(II) than Ca(II). The calculated geometries are shown in detail in Supplementary Table S5-7. Moreover, the EDX analysis and the element map (Supplementary Fig. S15) confirmed that PFCMP-0 adsorbed Pb(II), and the lead elements were distributed homogeneously inside and on the surface of the PFCMP-0 network. The FE-SEM images in Supplementary Fig. S16 indicated that the PFCMP-0 framework treated with Pb(II) still contained clusters of nanopores, reinforcing the viewpoint that the mechanism of interaction between the PFCMP-0 and the cations can be ascribed to physical adsorption.

To further understand the performance of PFCMP-0 for metal ions adsorption, a mixture solution in which the concentration of the Pb(II), As(V) and Ca(II) ions are all 5 mg L^−1^ was used for evaluation. The results show that PFCMP-0 can remove all 3 ions simultaneously and the Pb(II) ions are preferentially and nearly completely removed after adsorption. However, only 46.7% of As(V) and 19% of Ca(II) were adsorbed. The adsorption ability of PFCMP-0 in the mixture solution is in accordance with that in adsorbing single metal ion, indicating that there is competition between the different ions in the adsorption process.

In summary, a perfluorous conjugated microporous polymer with a large surface area and a large pore volume was synthesised. The obtained polymer shows superhydrophobicity, fast adsorption kinetics, excellent recyclability and strong adsorption ability for organic solvents and oils, dyes and metal ions, which shows promise as an excellent adsorbent material for use in water purification. PFCMP-0 is the first reported adsorbent that can efficiently adsorb the three types of primary pollutants in water simultaneously. The adsorption capacities of PFCMP-0 for Congo red, Pb(II) and As(V) are higher than for any previously described porous polymers. The regeneration and recyclability of the PFCMP-0 polymer are simple to perform. Because PFCMP-0 possesses all of these excellent adsorption performances, it may lead to rapid progress in making portable and economical water purification appliances.

## Methods

All of the experiments involving air- and/or moisture-sensitive compounds were performed using Schlenk techniques under argon. The NMR spectra were recorded on a BRUKER MERCURY-PLUS 400-MHz type (1 H, 400 MHz; ^13^C, 100 MHz) spectrometer. The chemical shifts were determined in ppm using TMS as an internal standard. The solid-state NMR spectrum was measured on a Varian Infinity-400 spectrometer. The ^1^H-^13^C CP/MAS NMR spectra of PFCMP-0 was recorded at a spinning speed of 8 kHz. The TGA analysis was performed using a DIAMONO TG-DTA analyser (DE Instruments) with an automated vertical overhead thermo balance. The samples were heated at a rate of 5 °C/min up to 800 °C under a nitrogen atmosphere. The nitrogen adsorption and desorption isotherms of the polymers were obtained from a Quantachrome QUADRASORB SI instrument, and its Brunauer-Emmett-Teller (BET) surface areas were calculated by a 6-point BET measurement over pressure ranges of 0.05 to 0.30 P/P_0_ at 77.3 K. The pore size distributions and volumes were derived from the desorption isotherm using the nonlocal density functional theory (NL-DFT). The morphology images of FCMP were obtained using a Quanta 200 FEG (FEI Company) cold Field Emission Scanning Electron Microscope (FE-SEM). An Oxford Instruments 7200 EDX was used to conduct the elemental analysis. The high-resolution morphology images were achieved using a high-resolution transmission electron microscope (HR-TEM, Tecnai G^2^ F30, FEI). The water CA measurement was performed on a contact angle meter (DSA100, Kruss Company, German), which was conducted by pinning the sample powder on a glass substrate to give a macroscopically smooth surface for the contact angle measurement. The UV–vis adsorption spectra were obtained by a Cary 5000 UV-vis-NIR spectrophotometer (Varian Company). The GC-MS analysis was performed on a GCMS-QP2010SE spectrometer. The IR spectrum was obtained using a Spectrum GX FTIR Spectrometer (PerkinElmer Inc.). The concentrations of the metal ions in the solution are detected by an Optima 7300 DV Inductively Coupled Plasma Emission Spectrometer ((PerkinElmer Inc.). All of the calculations were performed with the Gaussian 09 software package. For the geometry optimisations and frequency calculations, we used the LANL2DZ basis set. Because the experiment used water as the solvent, we also incorporated considerable solvent effects into the calculation. We estimated the solvation energies in terms of the PCM model with water as a solvent.

## Author Contributions

Y.R.X. synthesized the polymer FCMP-0 and performed adsorption experiments. W.T.T. carried out the theoretical calculations. D.W.Q. designed the research. All authors co-wrote the manuscript.

## Additional Information

**How to cite this article**: Yang, R.-X. *et al*. Extraordinary Capability for Water Treatment Achieved by a Perfluorous Conjugated Microporous Polymer. *Sci. Rep.* **5**, 10155; doi: 10.1038/srep10155 (2015).

## Supplementary Material

Supplementary Information
