# Supplementary material for: Extraordinary Capability for Water Treatment Achieved by a Perfluorous Conjugated Microporous Polymer
Source: Sci Rep. 2015 May 14;5:10155. doi: 10.1038/srep10155 (PMC4431420; doi:10.1038/srep10155)
Supplement: Supplementary Information [file srep10155-s1.doc]

Supporting Information

**Extraordinary Capability for Water Treatment Achieved by a Perfluorous Conjugated Microporous Polymer**

*Rui-Xia Yang, Ting-Ting Wang, Wei-Qiao Deng**

**Experimental section**

**Materials:** 1,3,5-Trifluoro-2,4,6-triiodobenzene was purchased from Nanjing Chemlin Chemical Industry Company (Nanjing, Jiangsu Province, China) and was used as received. The other reagents and solvents were obtained from Aladdin.

*1,3,5-Trifluoro-2,4,6-tris(trimethylsilanylethynyl)benzene:* This reaction was slightly modified from the previous report^54^. 1,3,5-Trifluoro-2,4,6-triiodobenzene (300 mg, 0.58 mmol), bis(triphenylphosphine)palladium(II)chloride (16.8 mg, 0.08 mmol), and copper(I)iodide (61.9 mg, 0.08 mmol) were added into a 50 mL two-neck rounded-bottom flask, after which a 15 mL anhydrous Et_3_N:THF(2:1) solvent mixture was added. The mixture was stirred for 30 min at room temperature under an argon atmosphere. Later, 0.49 mL trimethylsilylacetylene (3.48 mmol) was added dropwise. Then, the reaction mixture was stirred at 50℃ for 48 h, with the process of the reaction monitored by TLC. After cooling to room temperature, the precipitates were filtrated, and the solvent was removed via evaporation. The product was purified by column chromatography, eluting with petroleum ether to give a light yellow solid (130mg, 52.5%). ^1^H NMR (400 MHz): δ= 0.26 (s,27H).

*1,3,5-Trifluoro-2,4,6-triethynylbenzene^55^:* 1,3,5-Trifluoro-2,4,6-tris(trimethylsilanylethynyl) benzene (1.3 g, 3 mmol), ammonium fluoride (810 mg, 21 mmol), and tetrabutylammonium fluoride trihydrate (130 mg, 6 mmol) were dissolved in 20 mL tetrahydrofuran. The reaction mixture was stirred at room temperature for 3 h. The inorganic salt was filtrated, and the solvent was evaporated. The product was purified by column chromatography, eluting with petroleum ether. The pure product was a white powder (140 mg, 21.4%). EI^+^-MS: m/z=204 (100, [M^+^]). ^1^H NMR (400 MHz): δ=3.54 (s, 3H); ^13^C NMR (400 MHz): δ=164.5, 162.1, 107.2, 100.5.

*PFCMP-0:* The polymer was synthesised via Pd(II)/Cu(I)-catalysed homocoupling polymerization^56^. In a typical procedure, 1,3,5-trifluoro-2,4,6-triethynylbenzene (109 mg, 0.54 mmol), bis-(triphenylphosphine)palladium(II) dichloride (18.89 mg, 5 mmol%), and copper iodide (5 mg, 5 mmol%) were placed in a round-bottom flask. The solids were dissolved in the mixture of toluene (2.0 ml) and Et_3_N (2.0 ml). The reaction mixture was then stirred at 70 °C for 3 days under an argon atmosphere. The resulting polymer was then collected by filtration and washed with chloroform, acetone, water and methanol several times before being Soxhlet extracted with methanol for 3 days. The product was dried in vacuo at 100°C to give insoluble dark brown solids.

*HCMP-1:* The polymer was synthesized via Pd(II)/Cu(I)-catalysed homocoupling polymerization of 1,3,5-triethynylbenzene, as reported in reference^26, 53^.

*Adsorption of organic solvents and oils*: The weighted adsorbents were filled in a small piece of tube, of which both ends were blocked with little cotton. Then the weighted quantities of the FCMP-0 tubes were immersed in different organic solvents or oils. Adsorption of organic solvents and oils was carried out by capillary effect. After the upper polymer was infiltrated with the solvents, the tube was removed. Then the quantity of the treated tube with solvents adsorbed was weighed immediately to avoid evaporation of the adsorbed solvents or oils. The adsorption capacity values were obtained by measuring the mass of the dry sample and then the mass after the oil/solvent adsorption. The method emerged from the reference^11, 27^.

*Adsorption of dyes:* The adsorption isotherm was obtained by the addition of 20 mg FCMP-0 into 30 mL aqueous dye solutions of different initial concentrations. The mixture was stirred overnight to reach adsorption equilibrium. Subsequently, the FCMP was removed, and the collected solution was reserved to be analysed by UV-vis spectrophotometer.

*Adsorption of metal ions:* All of the experiments for the metal ion adsorption were performed by the addition of 20 mg FCMP-0 into 30 mL metal ion aqueous dye solutions of different initial concentrations. The mixture was stirred overnight to reach the adsorption equilibrium. Subsequently, the FCMP-0 was removed, and the collected solution was reserved to be analysed by ICP. Apart from the metal ions mentioned in the manuscript, the PFCMP-0 also show adsorption ability for other mental ions, such as Fe(III). The adsorption capacity of PFCMP-0 for Fe(III) is 117.3 mg g^-1^ when the initial concentration is 100 mg L^-1^, which is much lower than that of Pb(II).

The maximum adsorption capacities of FCMP-0 for the dyes and metal ions were calculated by the following Langmuir adsorption model:$Qe=QmbCe/(1+bCe)$, where Q_e_ and Q_m_ are the adsorption capacity and the maximum adsorption capacity of the metal ions at equilibrium, respectively, Ce is the concentration of the metal ions in the aqueous solution at equilibrium, and b is a constant.

*Preparation of the FCMP-0 treated sponge^27^:* A portion of sponge (0.6×0.6×0.6 cm^3^) was washed with distilled water and acetone several times and then immersed into a mixture of FCMP-0 in a chloroform solution. The FCMP-0 microgel particles were physically coated over the pores both inside and on the surface of the sponges after removal of the chloroform under low-pressure distillation.


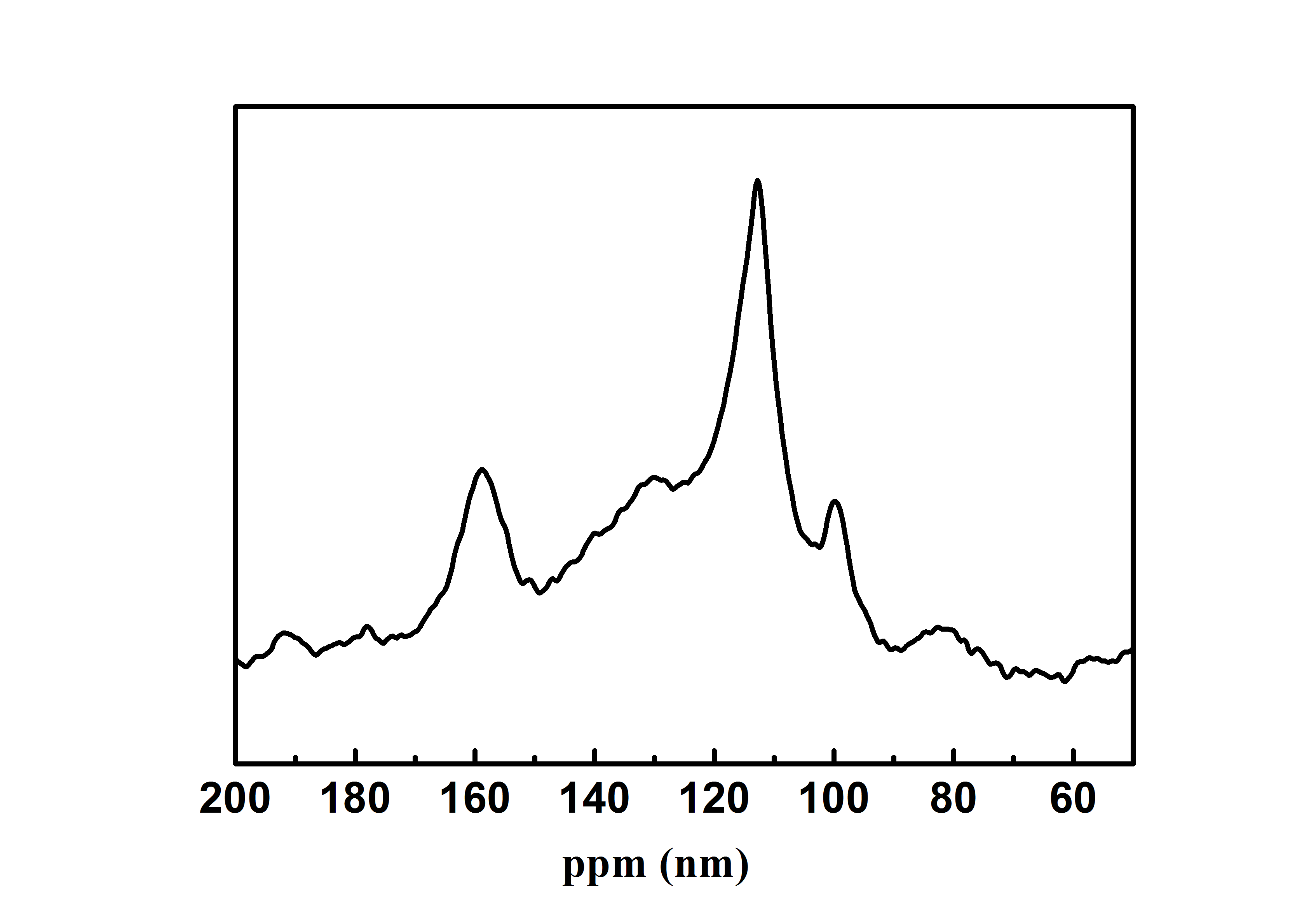


**Supplementary Figure S1.** Solid-state ^1^H-^13^C CP/MAS NMR spectra for PFCMP-0 recorded at an MAS rate of 8 KHz. The asterisks denote spinning sidebands. Solid-state NMR spectra were measured on an AVANCE III 500 DSX spectrometer operating at 100.61 MHz for ^13^C and 400.13 MHz for ^1^H. The assignment of the resonances was confirmed using ^1^H/^13^C CP/MAS kinetics and dipolar dephasing experiments. The solid-state NMR spectrum shows aromatic peaks at ca. 112 ppm, which can be ascribed to the aromatic carbons (C_Ar-C_), the peaks at ca. 97 and 81 ppm are due to the terminal alkinyl group functionalities and the peak at ca. 157 ppm was attributed to the aromatic carbons (C_Ar-F_). The chemical shifts of the carbons differ from the normal, indicating the intervention of fluorine.

**
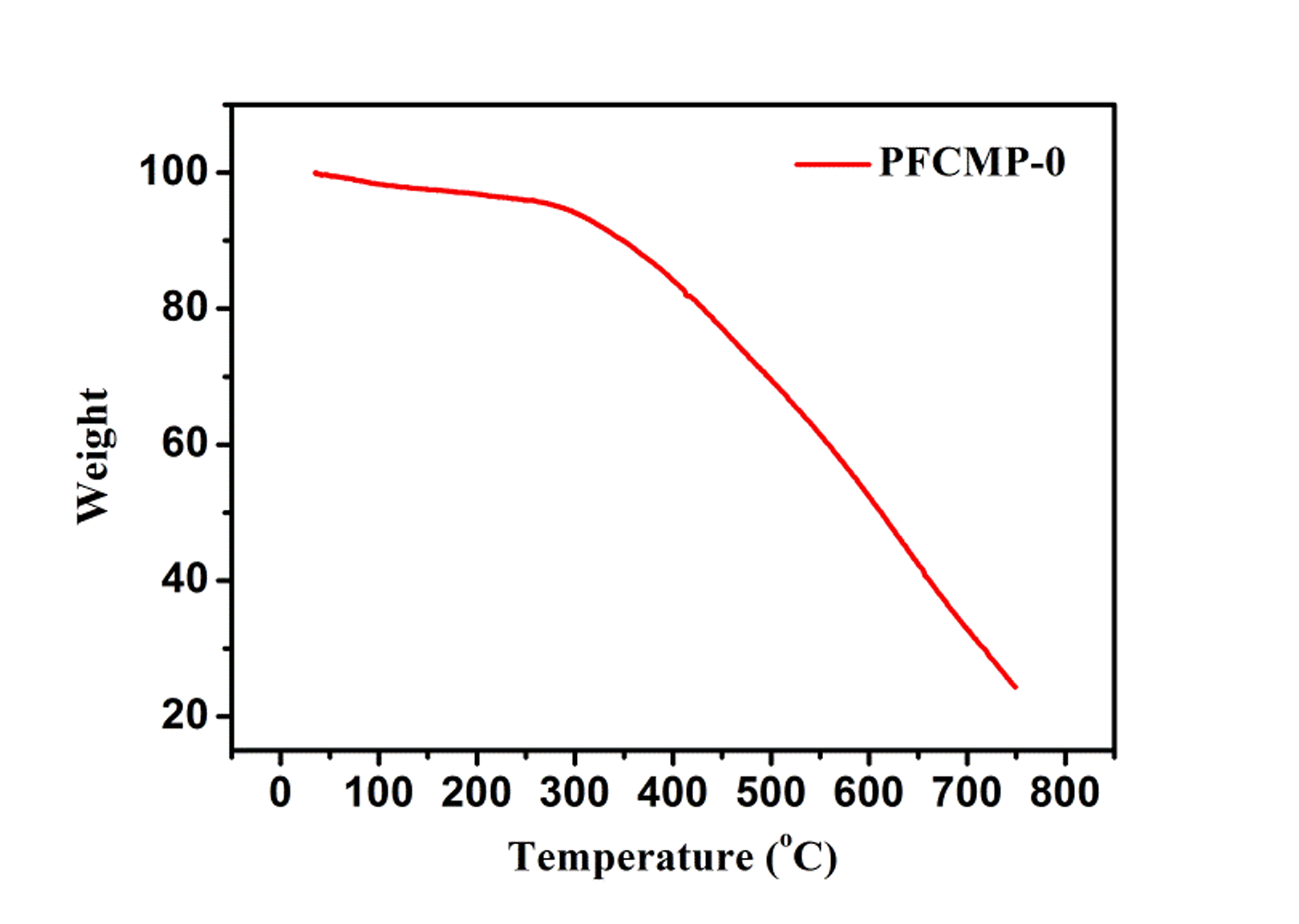
**

**Supplementary Figure S2.** The TGA curve of PFCMP-0. The thermal properties of the PFCMP-0 network was evaluated by thermogravimetric analysis (TGA) using a differential thermal analysis instrument (EXSTAR6000) over a temperature range of 50 to 700 °C under a nitrogen atmosphere with a heating rate of 5 °C/min.

**
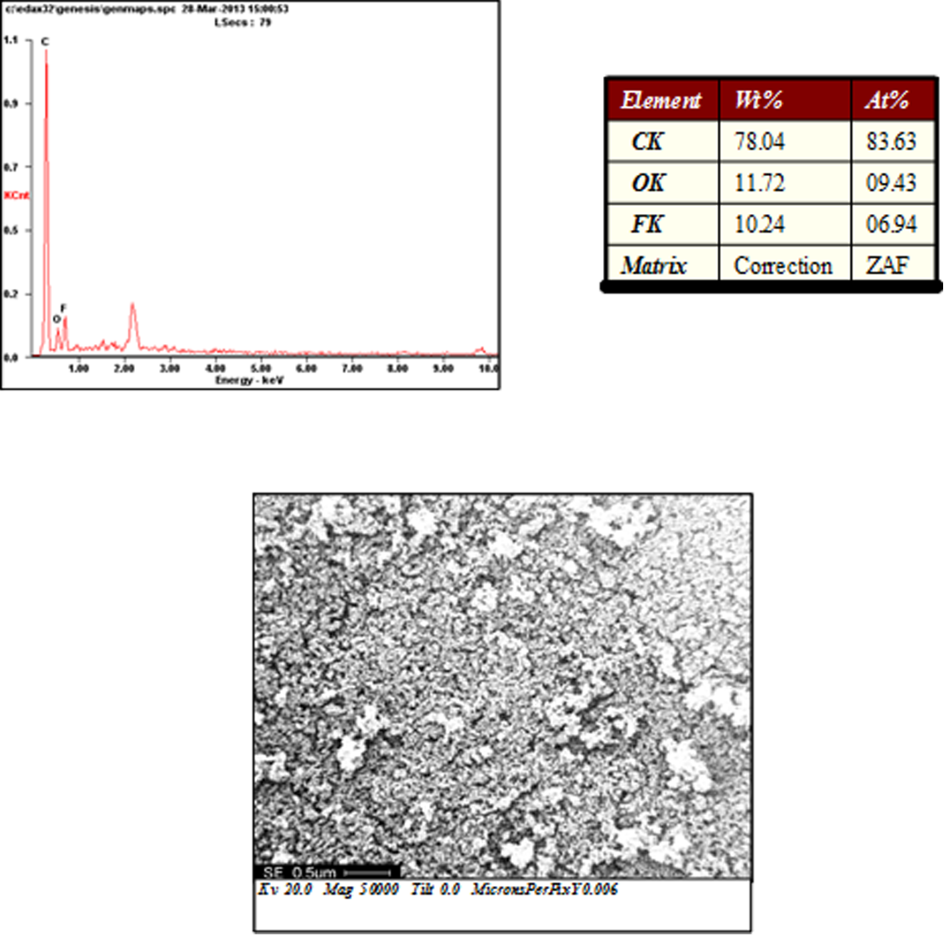
**

**Supplementary Figure S3.** The elemental distribution in the PFCMP-0 network. The EDX analysis shows that fluorine atoms are distributed throughout the polymer network, which is supported by the mapping results and element content provided in the table.

**
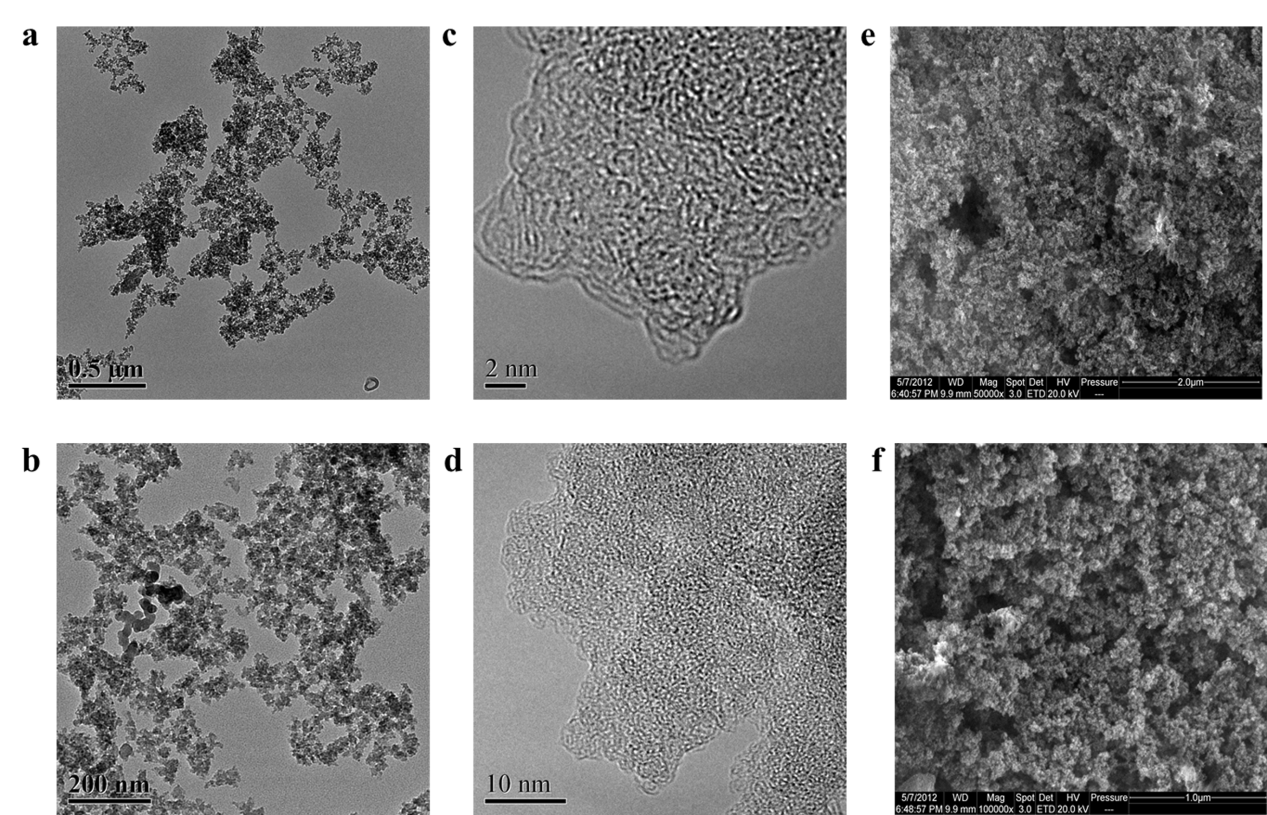
**

**Supplementary Figure S4.** HR-TEM photographs of PFCMP-0. (a) and (b)Transmission electron microscope (TEM) images of PFCMP-0 at different magnifications. (c) and (d) High-resolution transmission electron microscope (HR-TEM) images of PFCMP-0 at different magnifications. (e) and (f) Field emission scanning electron microscopy (FE-SEM) images at different magnifications. The sample used for field emission scanning electron microscopy was sputter-coated with gold prior to analysis. The sample used for the transmission electron microscope examination was ground, suspended in ethanol, and deposited on a copper specimen grid supported by a porous carbon film.


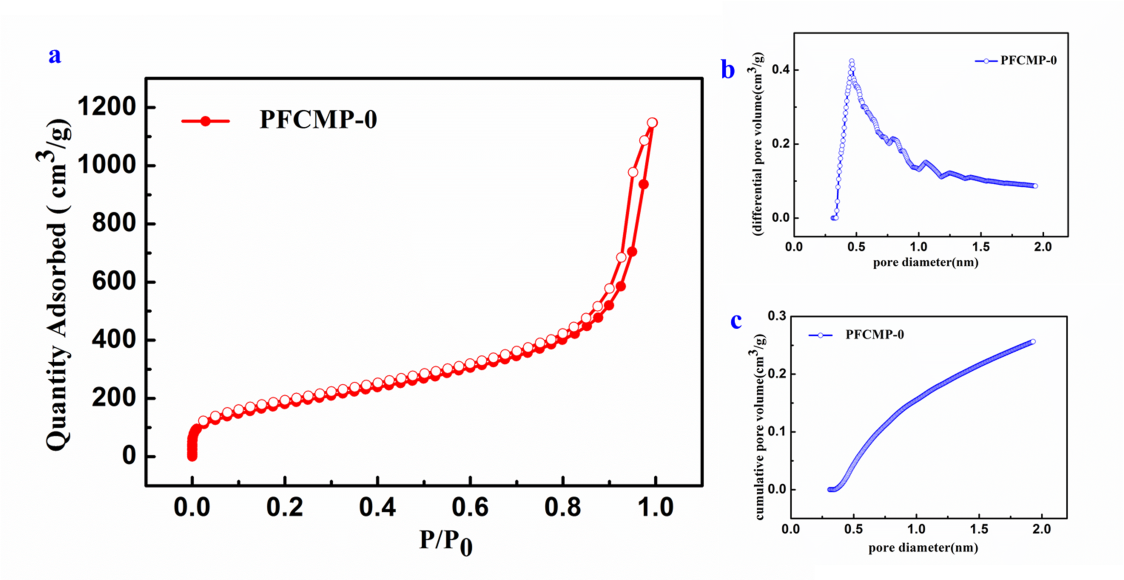


**Supplementary Figure S5.** The microporous properties of PFCMP-0. a) The nitrogen adsorption (filled) and desorption (unfilled) isotherms of PFCMP-0. b) The pore size distribution curve of PFCMP-0. c) The cumulative pore volume curve of PFCMP-0. The porous structures of the materials are characterised with nitrogen isotherms measured at 77 K using a pore and surface analyser.


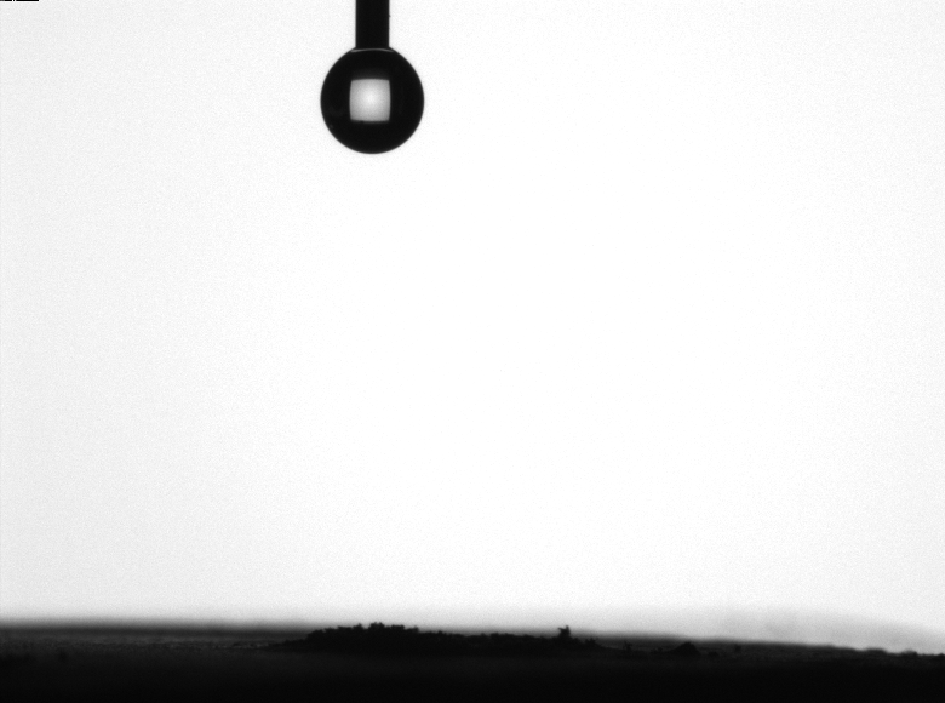


**Supplementary Figure S6.** The water CA for the active carbon. The water CA measurement was performed using a contact angle meter (DSA100, Kruss Company, German), which was conducted by pinning the sample powder on a glass substrate to give a macroscopically smooth surface for the contact angle measurement. The water contact angle of active carbon is 0°, which indicates that the active carbon is hydrophilic.

**
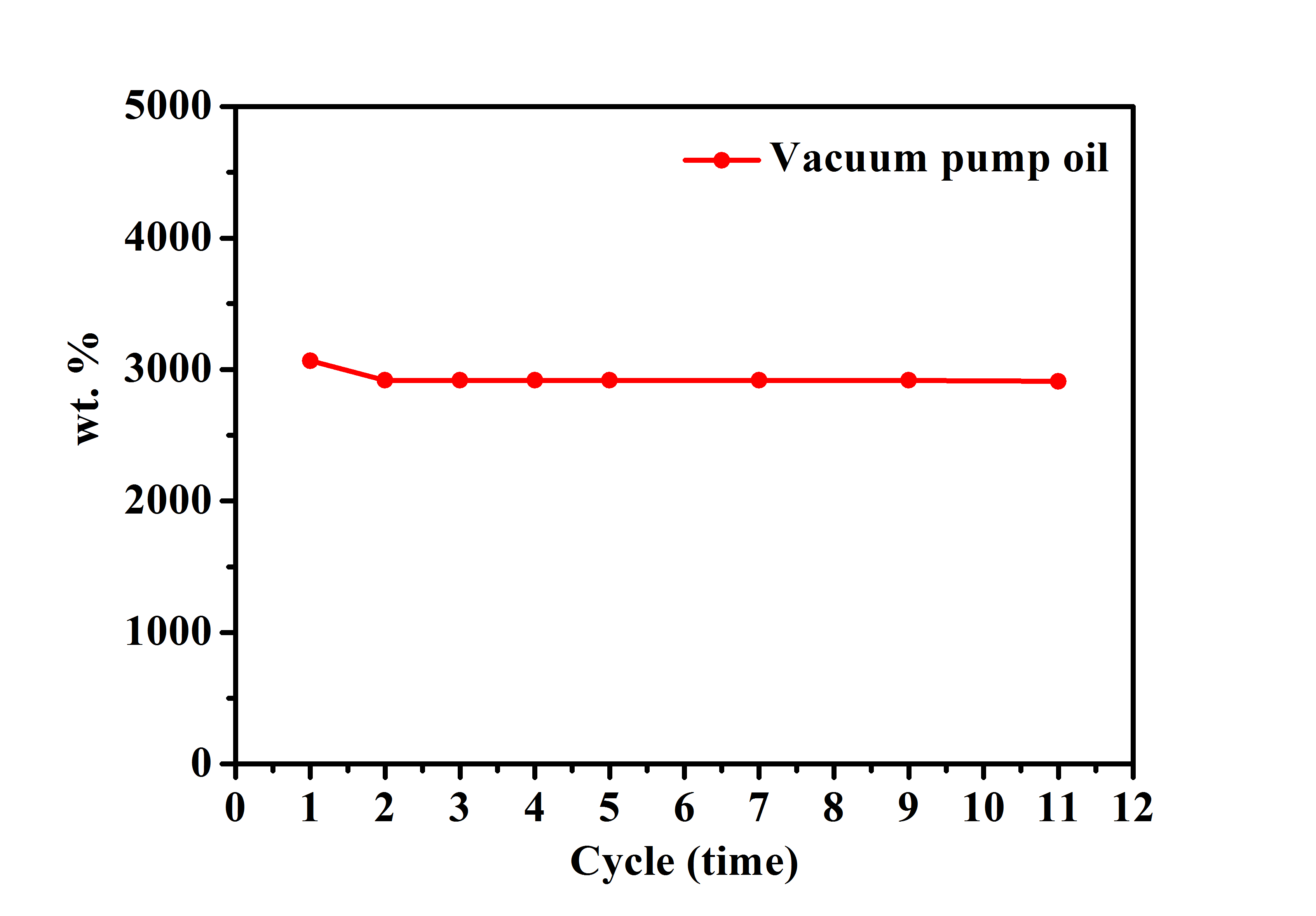
**

**Supplementary Figure S7.** Recycling of PFCMP-0 after adsorption of oils. Vacuum pump oil was chosen as representate to demonstrate that PFCMP-0 can be excellently regenerated and recycled.


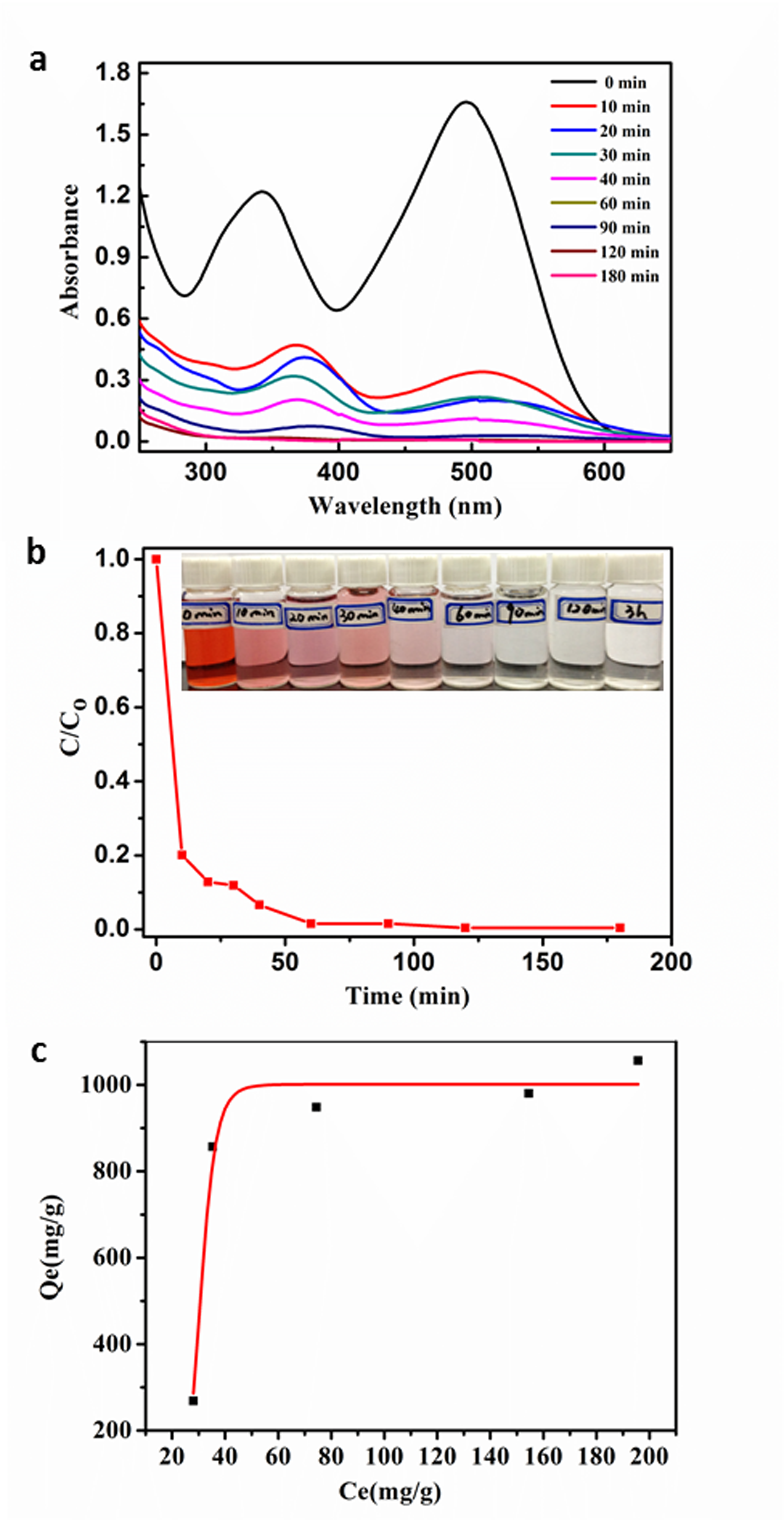


**Supplementary Figure S8.** Dye adsorption properties of HCMP-1. (a) UV–vis adsorption spectra of the CR anhydrous solutions after being treated at different intervals. The initial concentrations of the CR solutions are 100 mg L-1. (b) The adsorption rates of CR adsorption. The insets show the corresponding images. (c) The adsorption isotherms of CR as a function of their equilibrium concentrations.

**
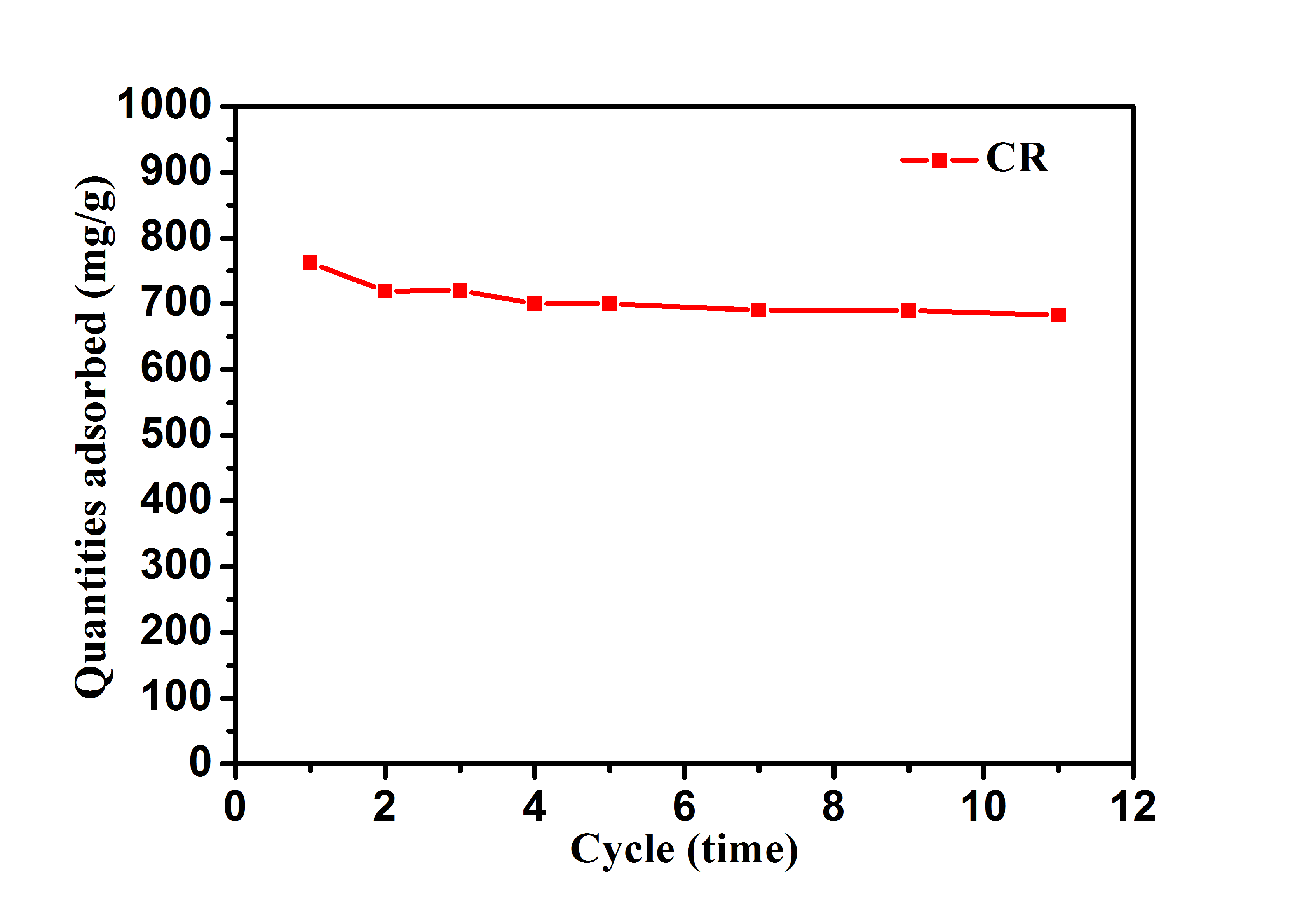
**

**Supplementary Figure S9.** Recycling of PFCMP-0 after adsorption of dyes. Congo red was chosen as an example, and the initial concentration of the CR solution was 100 mg


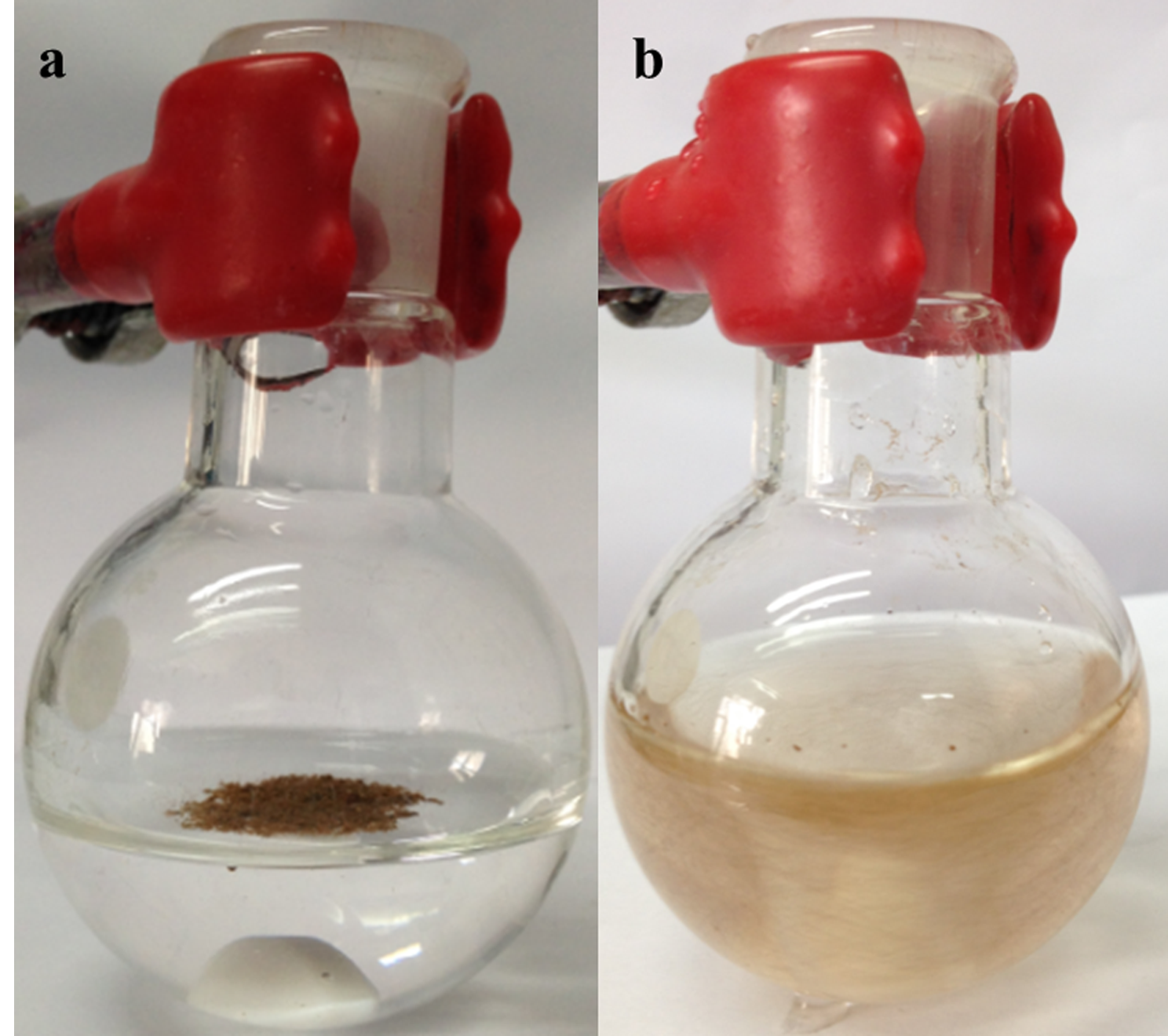


**Supplementary Figure S10** The images of the mixture of PFCMP-0 and the metal ions water solution. a) Because of its superhydrophobicity, PFCMP-0 floats on the surface of the metal ions water solution without stirring. b) PFCMP-0 dispersed in metal ions water solution under stirring condition.


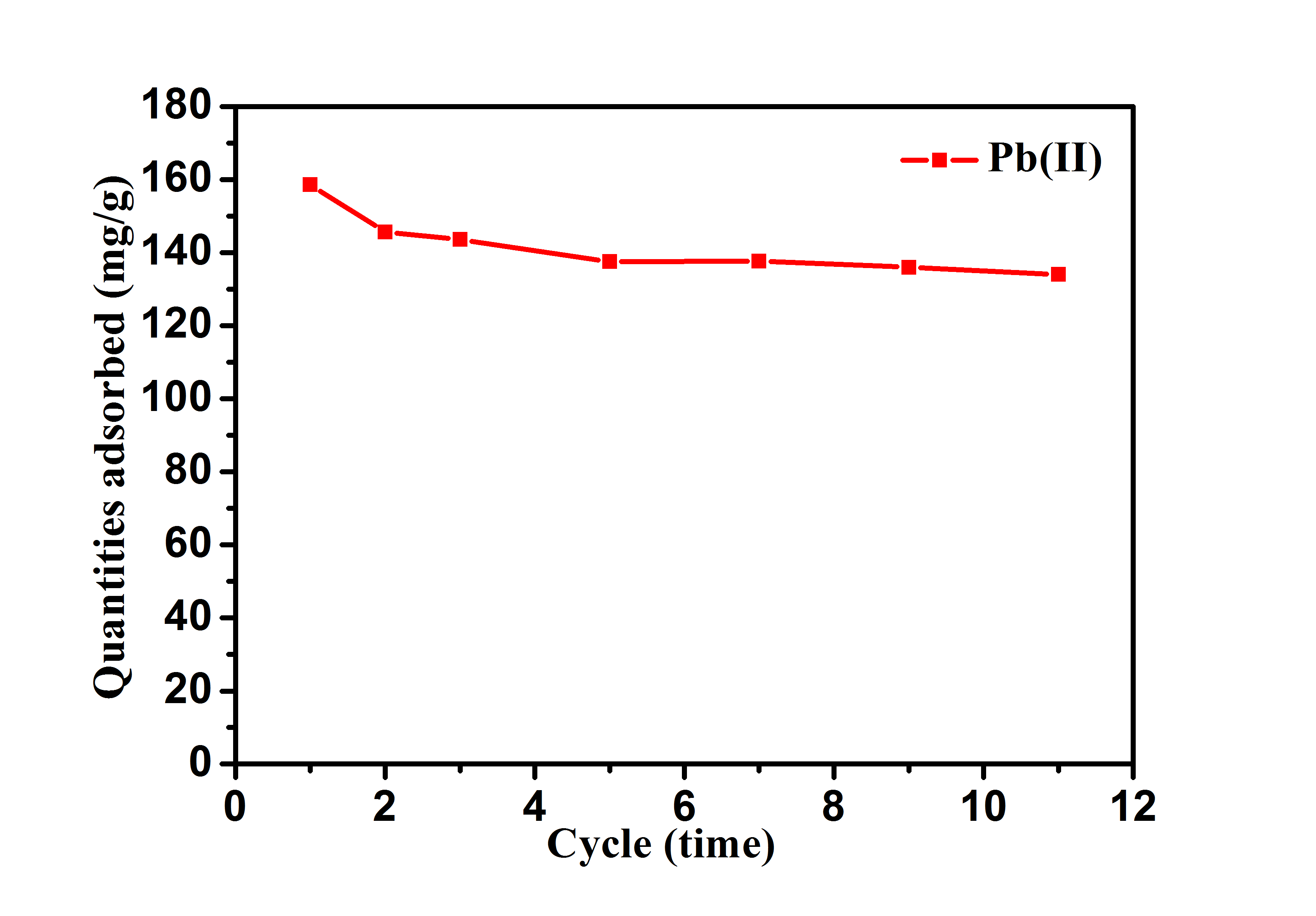


**Supplementary Figure S11. R**ecycling of PFCMP-0 after adsorption of metal ions. Pb(II) was chosen as an example, and the initial concentration of the Pb(II) solution was 50 mg L^-1^.

**
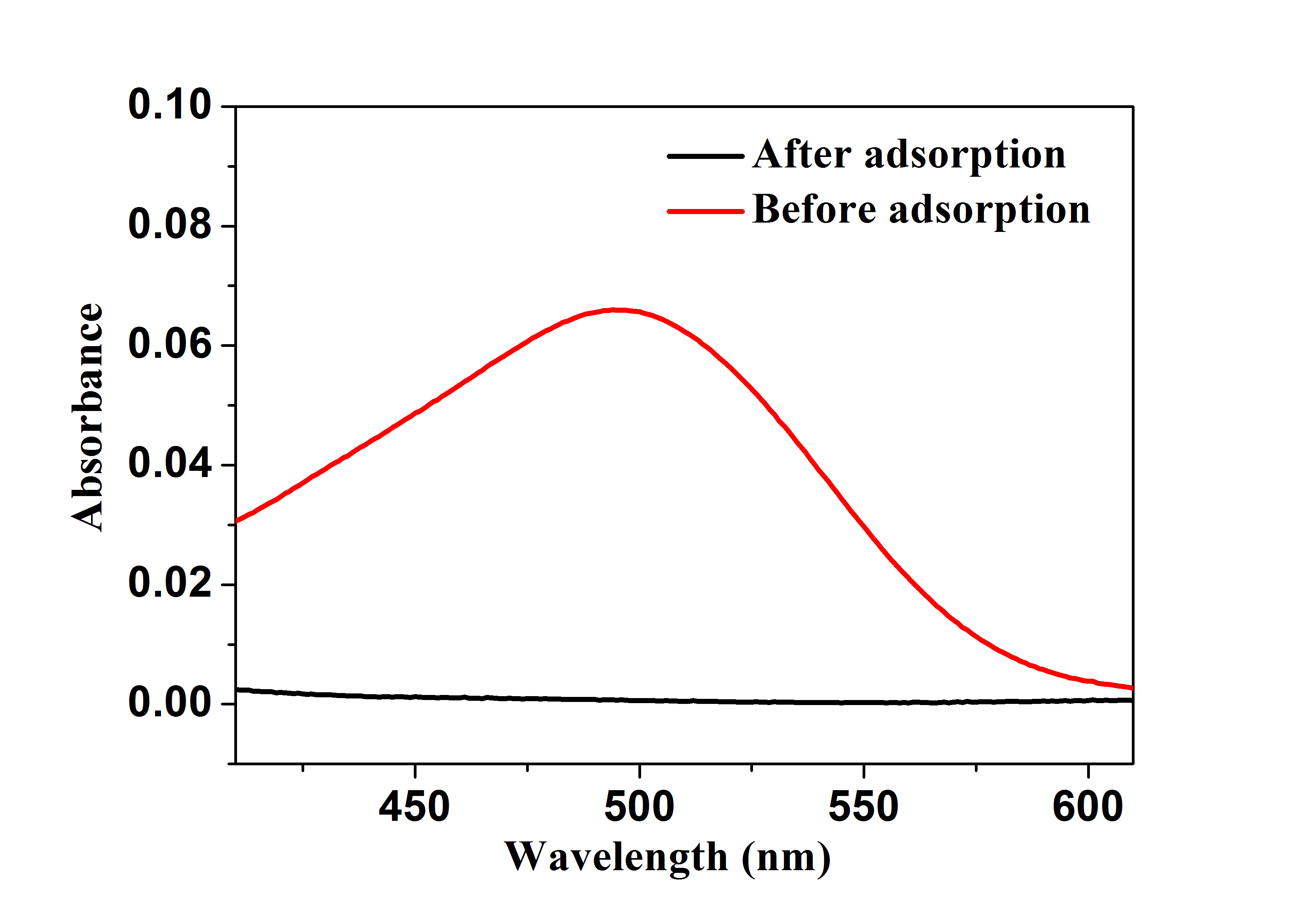
**

**Supplementary Figure S12.** UV–vis adsorption spectrum of the CR solution. The initial concentration of CR in the mixture of the three types of pollutants was 10 mg L^-1^.

**
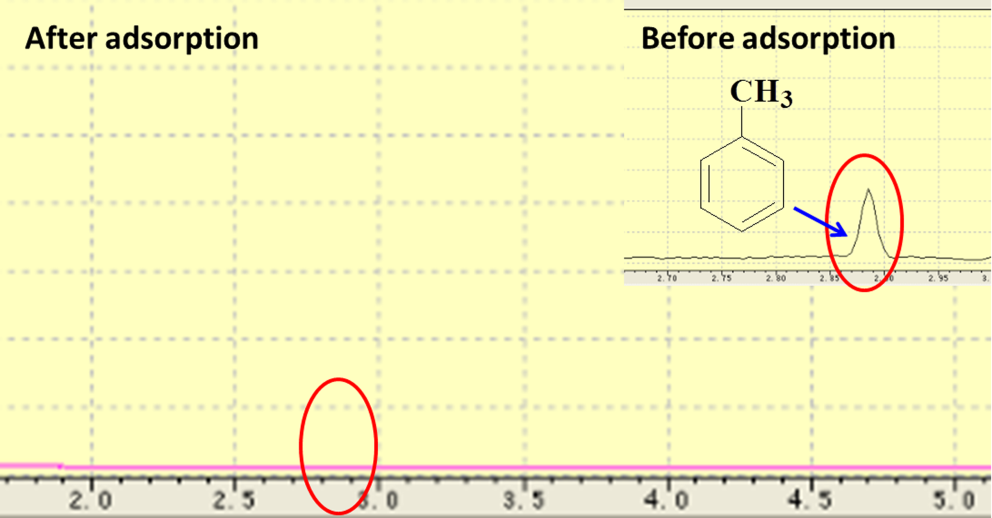
**

**Supplementary Figure S1****3.** The GC-MS spectrum of a mixed solution before and after treatment with PFCMP-0. The solution was mixed with CR (10 mg L-1), Pb(II) (5 mg L-1) and toluene (866 mg L-1). The inset shows that the retention time of the major peak of toluene appeared at 2.895 min; however, it disappeared after adsorption.

**
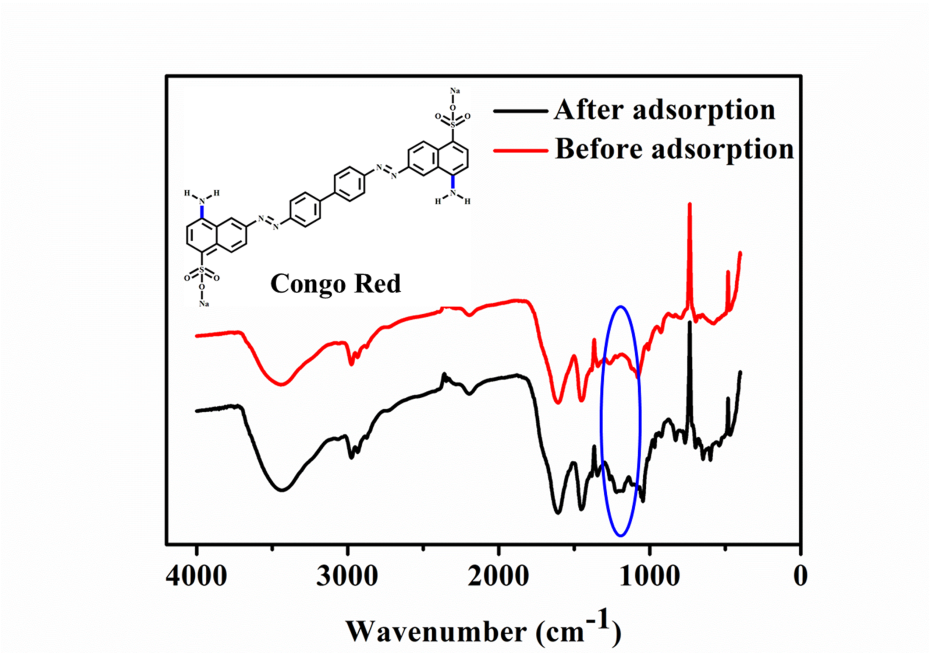
**

**Supplementary Figure S14.** FT-IR spectra of PFCMP-0 and PFCMP-0 with adsorbed Congo red.

**
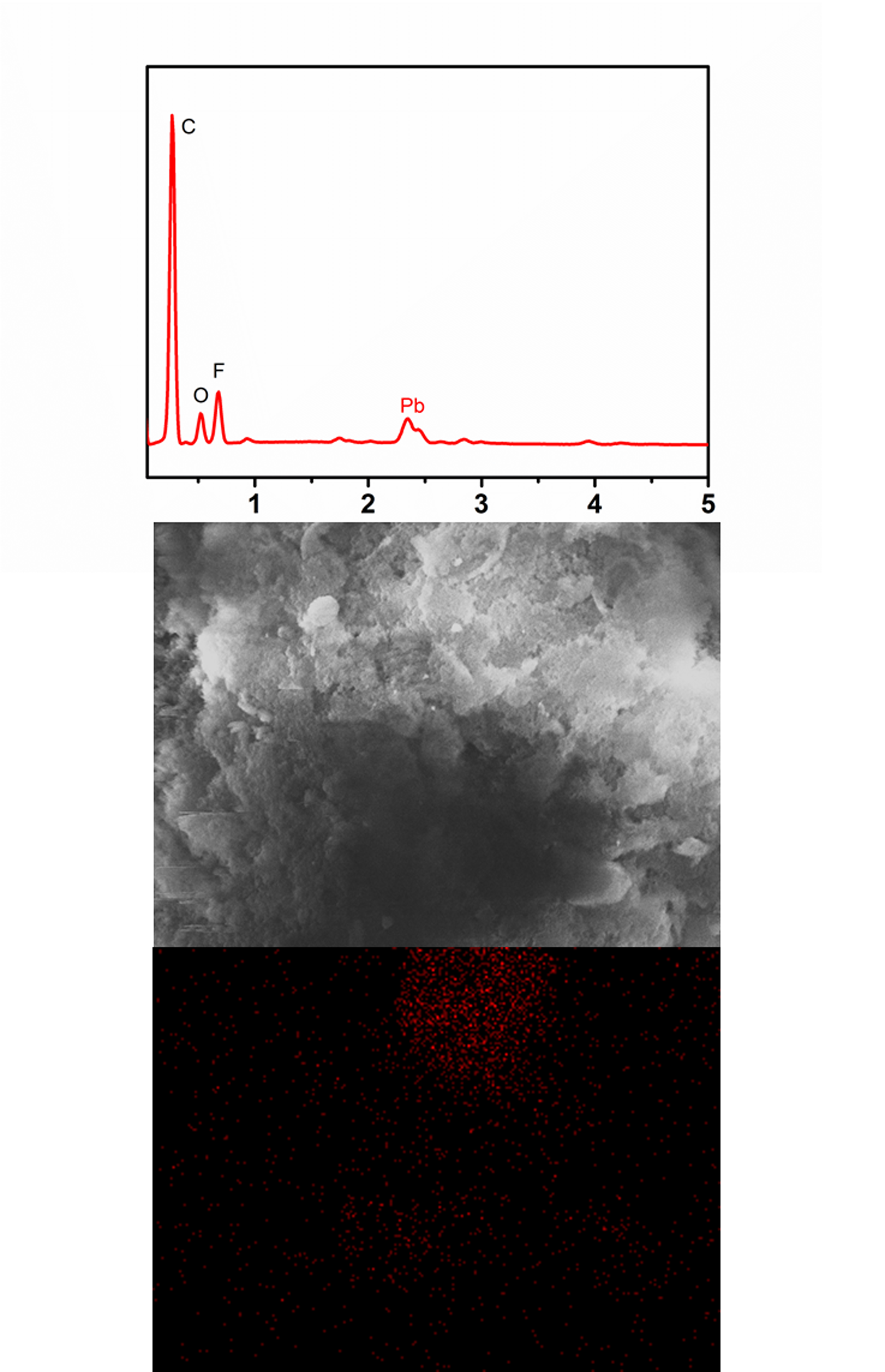
**

**Supplementary Figure S15.** The EDX analysis of PFCMP-0 with adsorbed Pb(II).

**
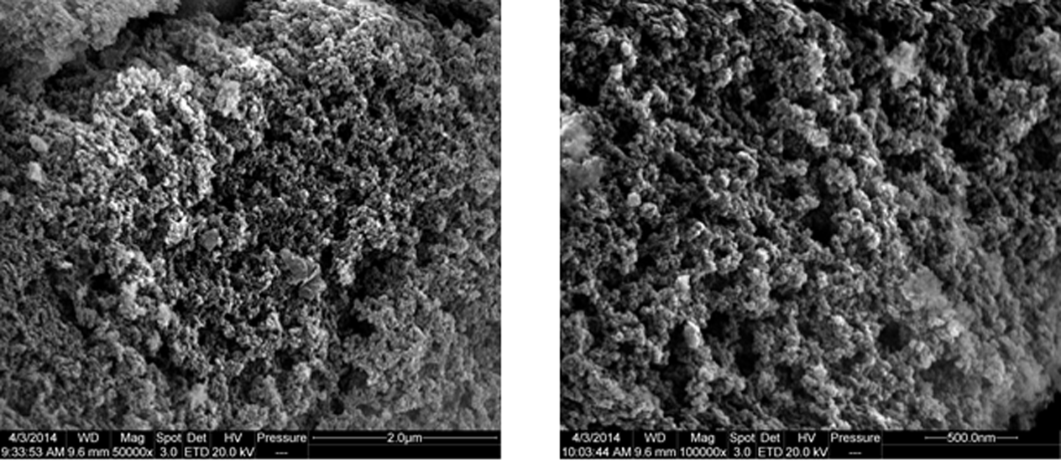
**

**Supplementary Figure S16.** Field emission scanning electron microscopy (FE-SEM) images of PFCMP-0 with adsorbed Pb(II) at different magnifications. The sample used for field emission scanning electron microscopy was sputter-coated with gold prior to analysis.

**Supplementary Table S1. Summarised data of the adsorption capacity (Q) of oil on a variety of materials.**

| **Absorbents** | **Q (wt %)** | **Reference** |
| --- | --- | --- |
| **PFCMP-0** | **3066** | **This study** |
| Boron nitride nanosheets | 3300 | 11 |
| PCF-1 | 2050 | 33 |
| HCMP-1 | 1100 | 26 |
| Nanowire membrane | 2000 | 8 |
| sponges | 1900 | 34 |
| graphene-based hydrogels | 1500 | 12 |

**Supplementary Table S2.** Summarised data of the maximum adsorption capacity (Qm) of dyes on a variety of materials.

| **Absorbents** | **CR**  **Q_max_(mg g^-1^)** | **MB**  **Q_max_(mg g^-1^)** | **Reference** |
| --- | --- | --- | --- |
| **PFCMP-0** | **1376.7** | **629.1** | **This study** |
| Boron nitride nanosheets | 782 | 313 | 11 |
| Carbon nanotubes | 882 | _ | 32 |
| CNF-280 | _ | 603.43 | 40 |
| α-FeOOH hollow spheres | 275 | _ | 43 |
| BNHSs C | _ | 116.5 | 42 |
| Activated Carbon | 500 | 400 | 39 |

**Supplementary Table S3. Summarised data of the maximum adsorption capacity (Qm) of Pb(II) on a variety of materials.**

| **Absorbents** | **Pb(II)**  **Q_max_(mg g^-1^)** | **Reference** |
| --- | --- | --- |
| **PFCMP-0** | **826.1** | **This study** |
| Carbonaceous Nanofiber Membranes | 423.7 | 40 |
| α-FeOOH hollow spheres | 80 | 43 |
| AAO-polyrhodanine membrane | 480.7 | 57 |
| CAS3 | 152.74 | 47 |
| starch graft copolymers | 433 | 58 |
| GNS-700 | 35 | 59 |
| hematite hollow spindles | 5.3 | 60 |
| pine cone activated carbon (PCAC) | 50 | 61 |
| Multiwalled Carbon Nanotubes | 50 | 62 |
| Activated Carbon | 21.8 | 44 |

**Supplementary Table S4. Summarised data of the maximum adsorption capacity (Qm) of As(V) on a variety of materials.**

| **Absorbents** | **As(V)**  **Qm (mg g^-1^)** | **Reference** |
| --- | --- | --- |
| **PFCMP-0** | **303.2** | **This study** |
| α-FeOOH hollow spheres | 58 | 43 |
| Fe_3_MOSF | 248 | 46 |
| Fe_3_O_4_ nanoparticles (12 nm) | 180 | 63 |
| Al(OH)CO_3_ nanospheres | 170 | 64 |
| α-Fe_2_O_3_ @ carbon | 29.4 | 65 |
| Fe_2_O_3_ CAHNs | 137.5 | 66 |
| 3D Flowerlike Iron Oxide Nanostructures | 7.6 | 67 |

**Supplementary Table S5.** Geometrical structure, energy and coordinates of PFCMP-0.


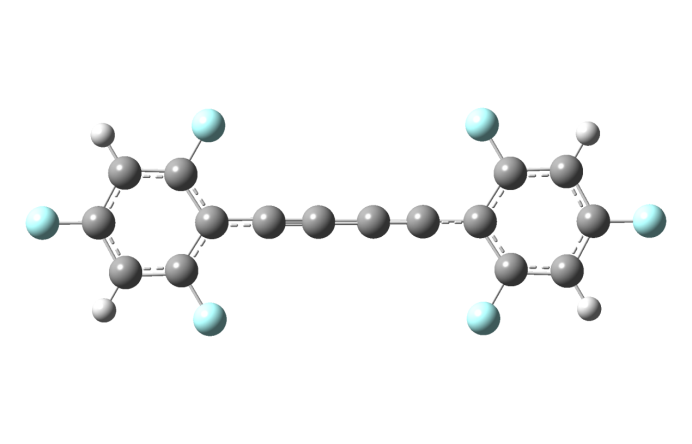


FCMP E= -1210.9779071 Hartree

C 5.48601300 1.23291800 0.00013400

C 4.09394000 1.19340800 -0.00004600

C 3.33370000 0.00015100 -0.00013800

C 4.09354700 -1.19335700 -0.00003700

C 5.48560800 -1.23332500 0.00014400

C 6.14674800 -0.00031200 0.00022600

H 6.02753000 2.17157100 0.00019900

H 6.02681600 -2.17215600 0.00021700

C 1.91361200 0.00039100 -0.00034000

C 0.68323000 0.00058800 -0.00048400

C -0.68323000 0.00058000 -0.00028400

C -1.91361200 0.00050700 -0.00015900

C -3.33370000 0.00020700 -0.00004600

C -4.09398900 1.19343300 0.00001600

C -4.09349800 -1.19333200 0.00000400

C -5.48606400 1.23288400 0.00012400

C -5.48555700 -1.23335800 0.00011400

C -6.14674800 -0.00037300 0.00017000

H -6.02761900 2.17151500 0.00016800

H -6.02672600 -2.17221200 0.00015100

F -3.39956800 2.40190900 -0.00003600

F -3.39858000 -2.40152200 -0.00005600

F -7.54182100 -0.00065700 0.00027800

F 3.39867900 -2.40157600 -0.00012700

F 3.39946900 2.40185600 -0.00014700

F 7.54182100 -0.00054000 0.00040700

**Supplementary Table S6.** Geometrical structure, energy and coordinates of Pb-PFCMP-0.


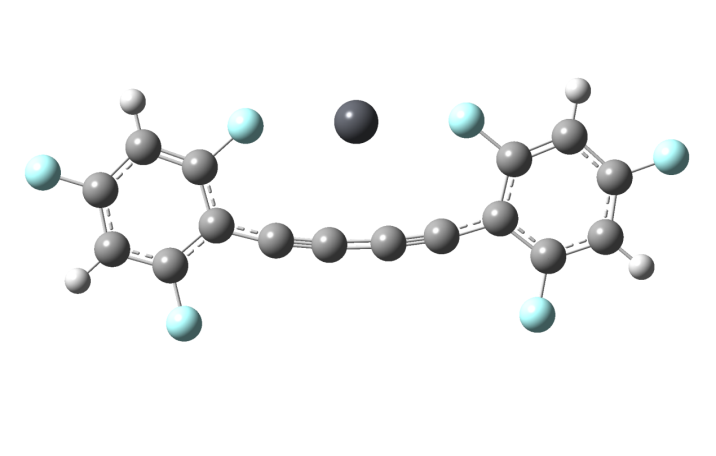


Pb-FCMP E= -1214.0918912 Hartree

C 5.69247400 1.52786800 0.03292600

C 4.36154300 1.93528700 -0.01660600

C 3.27417300 1.03720600 0.05198700

C 3.62747900 -0.32547000 0.17427400

C 4.92078400 -0.81415300 0.23019500

C 5.93401900 0.15469200 0.15617000

H 6.50437400 2.24363900 -0.02130700

H 5.13969700 -1.87115900 0.32244000

C 1.90888400 1.41227500 0.00176400

C 0.68241500 1.54561700 -0.01967700

C -0.68471200 1.54578200 -0.02038600

C -1.91166700 1.41609600 0.00183400

C -3.27624700 1.03838300 0.05195300

C -3.62729200 -0.32479600 0.17432400

C -4.36516000 1.93462900 -0.01675100

C -4.91978400 -0.81552100 0.23014100

C -5.69541500 1.52515500 0.03299200

C -5.93474300 0.15164300 0.15624200

H -5.13683400 -1.87288800 0.32222400

H -6.50851900 2.23956500 -0.02118700

Pb 0.00147300 -1.34035800 -0.16421100

F 2.55166300 -1.25603900 0.23739400

F 4.07403800 3.28625200 -0.13856300

F 7.25165100 -0.27848900 0.20866700

F -4.08005100 3.28615500 -0.13882000

F -2.55008400 -1.25402200 0.23766300

F -7.25166600 -0.28363100 0.20863800

**Supplementary Table S7.** Geometrical structure, energy and coordinates of Ca-PFCMP-0.


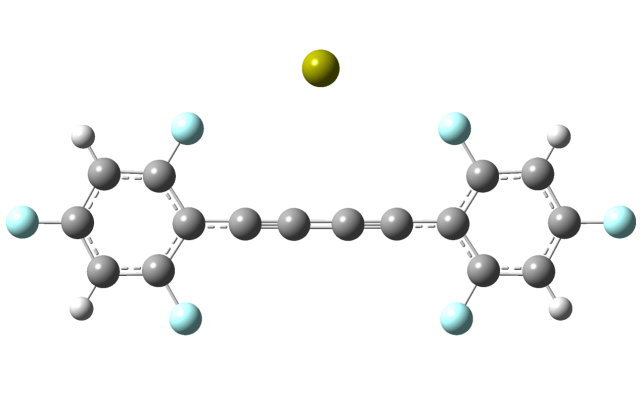


Ca-FCMP E= -1247.4192474 Hartree

C -5.49610000 -1.67203600 0.02182600

C -4.10362200 -1.64564600 0.02331500

C -3.33309200 -0.45950200 -0.01109200

C -4.08250800 0.73950300 -0.05076000

C -5.47354500 0.79353200 -0.05347600

C -6.14582800 -0.43338600 -0.01627800

H -6.04634000 -2.60524600 0.04867700

H -6.00633800 1.73663400 -0.08418000

C -1.91304000 -0.46920900 -0.01045400

C -0.68259600 -0.47762100 -0.00990200

C 0.68395700 -0.47881700 -0.01003000

C 1.91445200 -0.46964900 -0.01031700

C 3.33451500 -0.45934400 -0.01084200

C 4.08312600 0.74054400 -0.04600000

C 4.10589300 -1.64495200 0.01952500

C 5.47405700 0.79563800 -0.04948000

C 5.49845300 -1.67015600 0.01712500

C 6.14722000 -0.43098500 -0.01736500

H 6.00625500 1.73920000 -0.07628800

H 6.04941500 -2.60303800 0.04049800

Ca -0.00561500 3.46100200 0.11229700

F -3.37365300 1.94131800 -0.09335200

F -3.41970800 -2.85875300 0.06054700

F -7.54001200 -0.42002700 -0.01860500

F 3.42308500 -2.85874300 0.05274600

F 3.37347300 1.94215500 -0.07968000

F 7.54139700 -0.41651600 -0.02048000

**Supporting References**

54 Hennrich, G. & Echavarren, A. M. New persubstituted 1,3,5-trisethynyl benzenes via Sonogashira coupling. *Tetra. Lett.* **45**, 1147-1149 (2004).

55 Qu, J. Q., Zhang, J. Y., Grimsdale, A. C. & Mu1llen, K. Dendronized perylene diimide emitters: synthesis, luminescence, and electron and energy transfer studies. *Macromolecules.* **37**, 8297-8306 (2004).

56 Jiang, J. X. et al. Conjugated microporous poly(phenylene butadiynylene)s. *Chem. Commun.* 486-488 (2008).

57 Song, J., Oh, H., Kong, H. & Jang, J. Polyrhodanine modified anodic aluminum oxide membrane for heavy metal ions removal. *J. Hazard. Mater.* **187**, 311-317 (2011).

58 Zhang, L. M. & Chen, D. Q. An investigation of adsorption of lead(II) and copper(II) ions by water-insoluble starch graft copolymers. *Colloids and Surfaces A: Physicochemical and Engineering Aspects.* **205**, 231-236 (**2002)**,.

59 Huang, Z. H. et al. Adsorption of lead(II) ions from aqueous solution on low-temperature exfoliated graphene nanosheets. *Langmuir.* **27**, 7558-7562 (2011).

60 Zeng, S. Y. et al. Hematite hollow spindles and microspheres: selective synthesis, growth mechanisms, and application in lithium ion battery and water treatment. *J. Phys. Chem. C.* **111**, 10217-10225 (2007).

61 Momčilović, M., Purenović, M., Bojić, A., Zarubica, A. & Ranđelović, M. Removal of lead(II) ions from aqueous solutions by adsorption onto pine cone activated carbon. *Desalination.* **276,** 53-59 (2011).

62 Yu, X. Y. et al. Adsorption of lead(II) on O_2_-plasma-oxidized multiwalled carbon nanotubes: thermodynamics, kinetics, and desorption. *ACS Appl. Mater. Inter.* **3**, 2585-2593 (2011).

63 Yavuz, C. T. et al. Low-field magnetic separation of monodisperse Fe_3_O_4_ nanocrystals. *Science.* **314**, 964-967  **(**2006).

64 Cao, C. Y. et al. High adsorption capacity and the key role of carbonate groups for heavy metal ion removal by basic aluminum carbonate porous nanospheres. *J. Mater. Chem.* **22,** 19898-19903 (2012).

65 Wu, Z. X., Li, W., Webley, P. A. & Zhao, D. Y. General and controllable synthesis of novel mesoporous magnetic iron oxide@carbon encapsulates for effiient arsenic removal. *Adv. Mater.* **24**, 485-491 (2012).

66 Mou, F. Z. et al. Solvent-mediated synthesis of magnetic Fe_2_O_3_ chestnut-like amorphous-core/g-phase-shell hierarchical nanostructures with strong As(V) removal capability. *J. Mater. Chem.* **21**, 5414-5421 (2011).

67 Zhong, L. S.et al. Self-Assembled 3D flowerlike iron oxide nanostructures and

their application in water Treatment. *Adv. Mater.* **18**, 2426-2431 (2006).
